# Influence of Humic Acid Complexation with Metal Ions on Extracellular Electron Transfer Activity

**DOI:** 10.1038/srep17067

**Published:** 2015-11-23

**Authors:** Shungui Zhou, Shanshan Chen, Yong Yuan, Qin Lu

**Affiliations:** 1 College of Resources and Environment, Fujian Agriculture and Forestry University, Fuzhou 350002, China; 2 Guangdong Institute of Eco-environmental and Soil Sciences, Guangzhou 510650, China

## Abstract

Humic acids (HAs) can act as electron shuttles and mediate biogeochemical cycles, thereby influencing the transformation of nutrients and environmental pollutants. HAs commonly complex with metals in the environment, but few studies have focused on how these metals affect the roles of HAs in extracellular electron transfer (EET). In this study, HA-metal (HA-M) complexes (HA-Fe, HA-Cu, and HA-Al) were prepared and characterized. The electron shuttle capacities of HA-M complexes were experimentally evaluated through microbial Fe(III) reduction, biocurrent generation, and microbial azoreduction. The results show that the electron shuttle capacities of HAs were enhanced after complexation with Fe but were weakened when using Cu or Al. Density functional theory calculations were performed to explore the structural geometry of the HA-M complexes and revealed the best binding sites of the HAs to metals and the varied charge transfer rate constants (*k*). The EET activity of the HA-M complexes were in the order HA-Fe > HA-Cu > HA-Al. These findings have important implications for biogeochemical redox processes given the ubiquitous nature of both HAs and various metals in the environment.

Humic acids (HAs), which are ubiquitous in natural environments, are organic macromolecules primarily derived from the degradation of plant and microbialmaterials^1^. HAs are generally considered recalcitrant under anaerobic conditions, but it was recently discovered that HAs play a significant biological role. Numerous studies have indicated that HAs not only reduce the toxic effects of metals, hydrocarbons and other chemicals on microorganisms, but can also directly catalyze the oxidation of toxic compounds by both anaerobic and aerobic microorganisms^2^,^3^. This is accomplished through HAs acting as electron shuttles. They accept electrons from humic-reducing bacteria, such as *Geobacter sp*. and *Shewanella* sp., and then donate electrons to Fe(III) minerals^4^ or other spatially distant electron acceptors that are not directly accessible^5^. Their electron shuttling capacity has been mainly attributed to the enriched quinone groups in HAs that interconvert between the three oxidation states of quinone, semiquinone, and hydroquinone via reversible single-electron transfer reactions^6^. There is also evidence supporting the contribution of phenolic hydroxyl groups and N- and S-containing moieties to the electron transfer ability of HA^7^.

HA is the most widely encountered natural complexing ligands, which tend to chelate various metal ions present in the environment. In HA-M complexes, HA can change the properties of metal ions. For instance, metal solubility can be increased when metals are complexed with free HA or decreased when metals are scavenged by humic films on mineral surfaces^8^. As a result, heavy metal (e.g., Cu, Pb, Ag, and Cd) bioavailability and toxicity are affected^9^. A previous study revealed that HA-M complexes are involved in extracellular electron transfer (EET) processes. EET, which is defined as the process in which electrons produced via the oxidation of electron donors are transferred to the outer surfaces of cells to reduce an extracellular electron acceptor, is a ubiquitous process in soil ecosystems^10^,^11^. Lovely *et al.* demonstrated that Fe(III) bound in humic substances could accept electrons transferred from *Geobacter metallireducens*^12^. Zhang *et al.* reported that insoluble HA-Fe complexes could function as a solid-form electron mediator for anaerobic microbial dechlorination and microbial reduction of Fe(III) oxide, and the electron shuttle function has been proven to be attributed to the organically bound Fe fraction^13^. As discussed above, metal ion binding to HA is ubiquitous; therefore, a proper understanding of the electron transfer properties of HA-M complexes in microbial systems is highly desired. How the redox activity of HA is affected by the binding of metal ions and to what extent these observations depend on measurable structural parameters of the HA-M complexes remains unclear.

The objective of this study was to explore the redox-activity of HA-M complexes, and to determine their roles in microbial extracellular electron transfer. Three metals (Fe, Cu and Al) were chosen due to their ubiquity, complexation and redox reactivity, and *Shewanella oneidensis* MR-1 was used as a model EET microorganism^14^. Fourier transform infrared spectroscopy (FTIR), X-ray photoelectron spectroscopy (XPS) and the stopped-flow technique were applied to the structural and kinetic characterization of the as-prepared HA-M. Microbial Fe(III) reduction, biocurrent generation and microbial azoreduction experiments were conducted to observe the effects of the HA-M complexes on the EET of *S. oneidensis*.

## Results

### Characterizations of the HA complexation with metal ions

**Figure 1:**
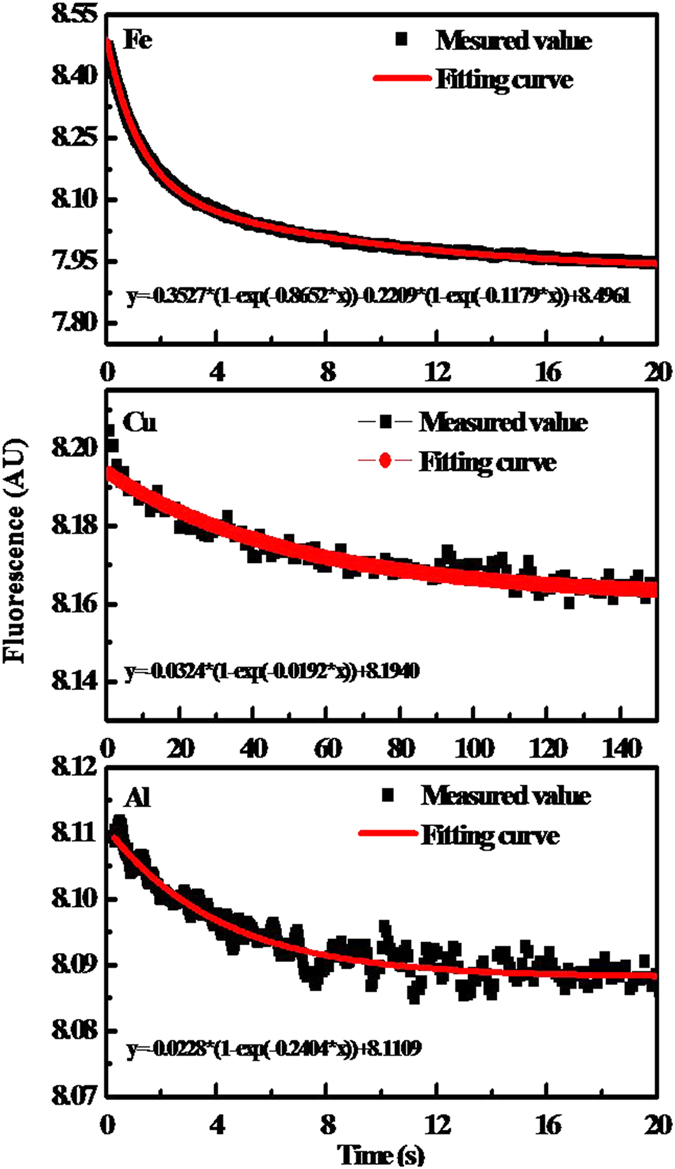
HA-M complexation kinetics and the corresponding fitting curves.

The fluorescence intensity as recorded by the stopped-flow spectrophotometer decreased rapidly as the reaction of HA-M complexation proceeded (Fig. 1), which was expected because previous reports showed that HA-M complexation occurs very rapidly, usually within tens of seconds^15^. Both HA-Fe and HA-Al complexation achieved equilibrium after approximately 20 s of reaction, while it took as long as approximately 150 s for the complexation between HA and Cu to achieve equilibrium. The kinetic data can be fitted with the following equation:


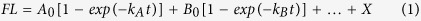


where *FL* is the fluorescence intensity, *A*_0_, *B*_0_, and *X* are constants, and *k*_A_ and *k*_B_ are the observed pseudo first-order rate constants. The fitting curves and the corresponding equations are shown in Fig. 1. As implied by the fitting curves, two major components in HA were simultaneously involved in the rapid complexation reaction with Fe (rate constants of 0.8652 and 0.1179 s^−1^) while only one major component in HA had participated in the HA-Cu or HA-Al complexation (rate constants of 0.0192 and 0.2404 s^−1^, respectively). The lower rate of complexation reaction of HA-Cu suggested weaker interactions in comparison with HA-Fe and HA-Al.

Elemental analyses showed that the pretreated HA had a C content of 64.74% and undetectable Fe, Cu, and Al, the HA-Fe complex had an Fe content of 263.2 mg/kg, the HA-Cu complex had a Cu content of 188.5 mg/kg, and the HA-Al complex had an Al content of 239.4 mg/kg (Table S1, Supporting Information). HA and the three HA-M complexes displayed very similar FTIR spectra with characteristic transmission peaks at approximately 3444, 1635, 1385, 1141, and 624 cm^−1^ (Fig. S1, Supporting Information). These peaks can be assigned to the OH stretching of phenolic OH, aromatic C=C stretching and/or asymmetric –COO-stretching, symmetric –COO-stretching and/or –CH bending of aliphatics, aliphatic –OH, and aromatic C-H bending vibrations, respectively according to the literature^16^. However, there are discernible differences in the relative peak intensities (Table S2, Supporting Information) which can be attributed to metal effects^17^, suggesting complexation between metals and HA bridged by functionalities such as –COO- and –OH^18^.

**Figure 2:**
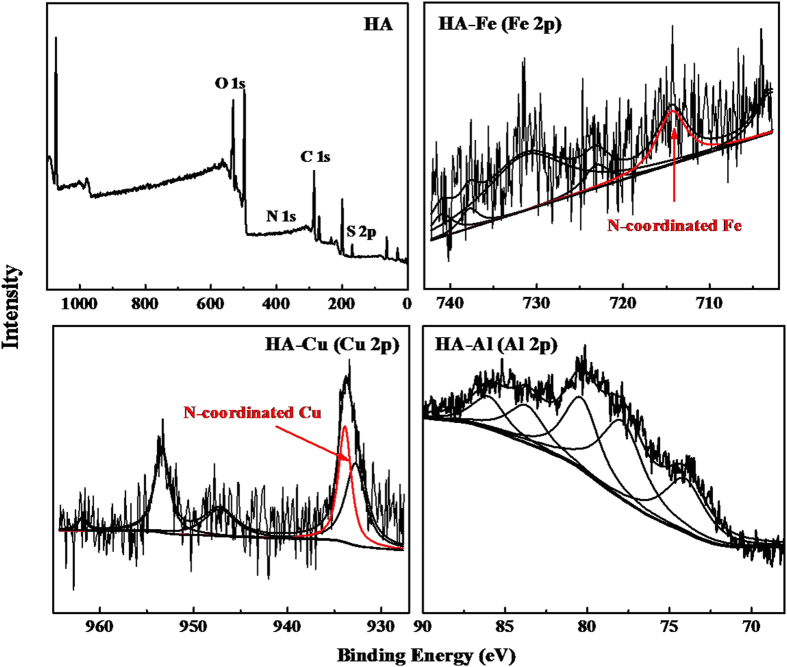
Survey XPS spectrum of HA and high-resolution Fe 2p, Al 2p, and Cu 2p XPS spectra of HA-M complexes.

High-resolution C 1 s XPS spectra showed that the majority of C in HA existed as C-H or C-C with a small amount present as O-C=O (Fig. S2, Supporting Information). The spectra of the HA-M complexes exhibited a new peak at about 286 cm^−1^ that did not appear in the original HA spectrum, thus indicating that C-O played a role in metal complexation. Deconvolution of the N 1 s spectra produced at least three peaks, indicating N in HA or HA-M complex present in multiple states including pyridinic N (398 eV), pyrrolic N (400 eV), and quaternary N (403 eV) (Fig. S3, Supporting Information). Pyridinic N atoms can be important metal coordination sites due to their electron-donating properties^19^. A prominent peak at 169 eV was displayed in the S 2p XPS spectra of the HA and HA-M complexes (Fig. S4, Supporting Information). Another peak at approximately 175 eV also appeared in the spectra of HA-Fe and HA-Al, which suggested the binding of S to metals, because previous studies found that the binding of S to metal would shift the **S**2p binding energy peaks by approximately 5 eV to higher energy^20^. The Fe2p XPS spectrum (Fig. 2) of the HA-Fe complex indicated the presence of both Fe^2+^ (722.9 eV and 730.7 eV) and Fe^3+^ (714.3 eV), and according to a previous report, the peak at 714.3 eV indicated N-coordinated Fe^21^. The peak at 933.8 eV in the Cu 2p XPS spectrum suggested the coordination bonding between Cu and the nitrogen of pyridine^22^. The Cu 2p XPS spectrum evidenced both Cu^+^ (932.8 eV) and Cu^2+^ (933.9 eV) in the HA-Cu complex, which confirmed that metals may be simultaneously redox-transformed when complexed with HA^23^. In addition, Cu (I) was very likely to form strong complexes with reduced organic sulfur due to its higher thiophilicity^24^. However, Al was only in the oxidized state of Al^3+^ (74.2 eV) in the HA-Al complex. The above experimental results suggested that the three metals had been successfully complexed with HA and that the C-O, N- and S-containing groups may play an active role in chelating the metals.

### Electron transfer properties of the HA-M complexes

**Figure 3:**
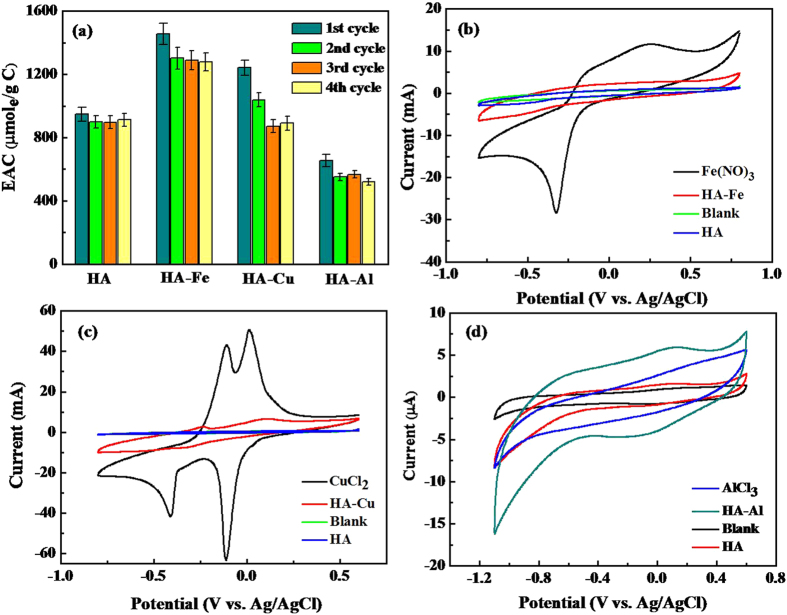
Electron transfer reversibility of (**a**) HA and HA-M complexes (Significant difference level (p < 0.05) was signed by using a superscript on the data bar). Cyclic voltammograms of HA-Fe (**b**), HA-Cu **(c**), and HA-Al (**d**) scanned in the potential range +0.6 to –0.8 V v.s. Ag/AgCl at 10 mV/s.

In terms of EAC (Fig. 3a), both the resulting HA-Fe and HA-Cu complexes had significantly higher EAC than did the original HA at the first cycle, while the EAC of HA was lowered after complexing with Al. This may be related to the redox activity of the three metals. Fe and Cu are substantially more redox active than Al. They showed well-defined oxidation and reduction peaks on cyclic voltammograms (Fig. 3b,c), whereas no obvious oxidation-reduction peaks were produced by Al (Fig. 3d). The binding of the metals by HA deceases the redox behavior of these metal ions compared to when they are free ions, which resulted from because the coordination bond was more stable than the ionic bond. The decrease in the EAC of HA after complexation with Al may result from a reduction in its available redox-active functionalities due to a blockage by Al. Compared with EAC, electron transfer reversibility is a more important feature for a redox mediator, which indicates the actual normalized amount of electrons participating in reversible electron transfer. Figure 3a shows that after 4 cycles, a stable electron transfer capacity was obtained for the HA and HA-M complexes, and the number of electrons measured in the 4th cycle was the portion of the electrons that can be reversibly transferred and thus is the material basis of HA and HA-M complexes as mediators. HA-Fe contains the highest number of electrons (1275 μmol_e_/g C) that can reversibly transfer electrons, while HA-Al has the lowest number of such electrons (522 μmol_e_/g C). Although the HA-Cu complex (1246 μmol_e_/g C) had substantially more electrons involved in the 1st cycle of electron transfer compared to HA (954 μmol_e_/g C), only approximately 71% (889 μmol_e_/g C) of the electrons can be recycled in HA-Cu, while as many as 94% (900 μmol_e_/g C) of the electrons can be reversely transferred in HA.

### Extracellular Electron Transfer Mediated by the HA-M Complexes

**Figure 4:**
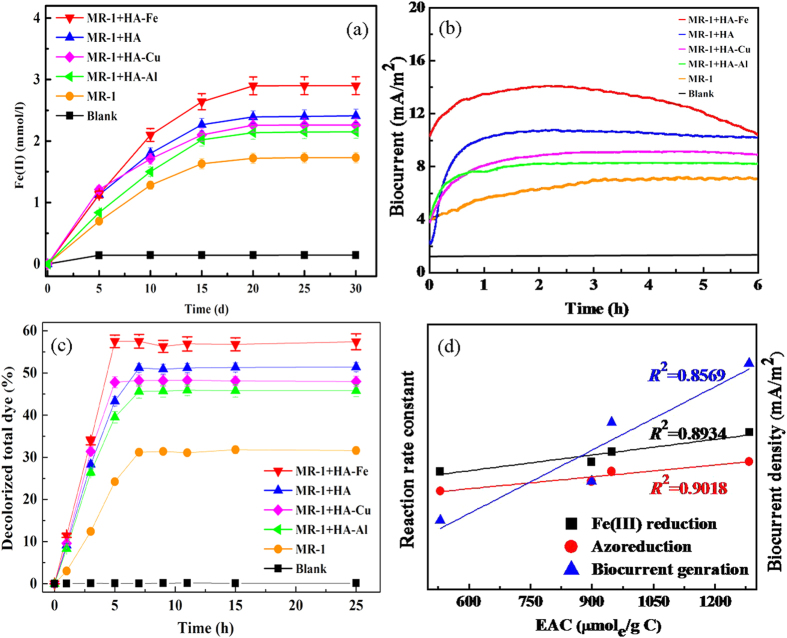
Extracellular electron transfer mediated by HA-M complexes. (**a**) Dissimilatory Fe(III) reduction, (**b**) biocurrent generation, (**c**) azoreduction, and (**d**) relationship between EAC and reaction rate constant.

The results of the Fe(III) reduction, biocurrent generation and azoreduction experiments with *S.oneidensis* MR-1 showed that the magnitudes of the accelerating effects or shuttling activities were in the order HA-Fe>HA > HA-Cu > HA-Al > blank (Fig. 4a–c). *S.oneidensis* MR-1 is an important dissimilatory metal-reducing bacterium (DMRB) and is able to use a variety of anaerobic terminal electron acceptors, including solid-phase iron oxides such as goethite and hematite. However, Fe(III) oxides are poorly soluble in circumneutral environments. Microbial reduction of Fe(III) oxides has to address a practically insoluble electron acceptor. Electron shuttles (HA and HA-M complex), by alleviating the need for Fe(III)-reducing microbes to establish direct contact with the Fe(III) oxides to reduce them, can accelerate Fe(III) reduction (Fig. 4a). At the later stage of the experiment, the Fe(II) concentration in all of the treatments reached a plateau, which was related to a substrate shortage and loss of microbial activity. In the generation study, the bioelectrochemical systems with added HA or HA-M complex generated a higher current compared with the MFC without HA or HA-M complex (blank) (Fig. 4b). This can be attributed to the electron shuttling role of these substances, which accelerated electron transfer and resulted in a higher current. Although the HA-Fe system produced the highest current for the first few hours, it was also the first to display decreased biocurrent generation, which may be explained by electron donor limitations caused by rapid consumption. The results shown in Fig. 4c also confirmed the positive effects of electron shuttles (i.e., HA and HA-M complexes) that accelerate electron transfer and drive azoreduction to proceed further before the growth of bacteria enters a less active stage and also before the bacteria are intoxicated by the toxic intermediates of orange I degradation. Orange I is one of the azo dyes, which are toxic, highly persistent, and ubiquitous in the environment^25^. Anaerobic bacterial azoreduction is an important pathway for azo dye degradation, but decolorization often proceeds very slowly^26^. Significantly increased reduction rates in systems with electron shuttles added have been reported^27^. The magnitudes of the accelerating effects of the HA and HA-M complexation electron shuttles were in the same order as those observed in the Fe(III) reduction and azoreduction experiments. After approximately 6 hours, decolorization rates in all of the systems reached a plateau, thus indicating that no additional orange I was degraded, which may be because of electron donor limitations or microbial toxication. Sterile controls of the microbial Fe(III) reduction, biocurrent generation and microbial azoreduction experiments were shown in Fig. 4, which demonstrated that no Fe(III) was reduced, no current was generated and no dye was decolored without the microbes. It should be mentioned that the HA and HA-Metal complexes had limited influence on the growth of *S. oneidensis*, which excluded the impact of microbial metabolism on the EET in the presence of HA and HA-Metal complexes.

## Discussion

HA includes electron shuttles can mediate microbial extracellular respiration. The above Fe(III) reduction, biocurrent generation, and azoreduction experiments demonstrate that after complexation with metals, the role of HA as an electron shuttle in extracellular electron transfer may be enhanced or weakened. The results consistently showed that the HA-Fe complex acted as an electron shuttle, accelerating Fe(III) reduction, biocurrent generation, and azoreduction by microbes. In contrast, the incorporation of Cu or Al weakened the role of HA as an electron shuttle. These results were in agreement with the previously measured electron reversibility. Specifically, the HA-Fe complex includes more electrons that can be reversibly transferred compared to HA, while the HA-Cu and HA-Al complexes have fewer electrons that can be reversibly transferred compared to HA (Figs 4a and 2d). The different effects of these three metal ions on the role of HA as electron shuttles may be related not only to the redox properties of the metals themselves but also to the nature of HA-M interactions (Scheme S1, Supporting Information). Fe is very redox active, and as a hard metal, its coordination to HA functionalities is mainly electrostatic in nature^24^,^28^, forming an outer-sphere complex with HA^7^. Therefore, complexed Fe is expected to be able to participate in electron transfer, as extensively documented in the literature^29^,^30^. Therefore, its incorporation produced additional redox active sites in HA, thereby enhancing the role of HA as electron shuttles^7^,^12^. Though also redox active, as a soft metal, Cu mainly forms very weak covalent bonds with HA^28^, which may hinder free electron transfer. Aluminum is not redox active; therefore, its connection with HA molecules not only produces no additional redox active sites but may also block certain redox active sites of the HA molecule. Consequently, the role of HA as electron shuttles is weakened after complexation with Al.

To further support our interpretation, the proposed structures of HA-M complexes were studied through DFT calculations. Given the complicated and unresolved structure of HAs, a model compound of low molecular weight was used to mimic HAs as ligands and to provide a general insight into the molecular and electronic structure of HA-M complexes. As suggested by Niederer *et al.*^31^, the HA monomer described in Fig. S5 and Table S4 (Supporting Information) was utilized as the model compound for the theoretical calculations and includes 8 possible active metal-binding sites (a ~ h). To simplify the calculations, only oxygen functional groups, such as carboxyl, phenol and carbonyl, were considered as the ligands for the metal complexation in this model compound. Although the N and S functional groups were also confirmed via XPS for complexation with metal ions, the N and S group contents were substantially lower than that of the O groups. Therefore, the N and S groups do not need to be considered in the model compound. The calculated energy of the individual metal ions and HA are listed in Table S2 (Supporting Information), from which the bond dissociation energies (ΔE) and energy spin states of the metals bonded with HA at various active sites were calculated. These results are presented in Table S3 (Supporting Information). The HA-M complexes exhibited the highest ΔE values under various spin states when the metals bonded with HA at active site d, thus suggesting that site d was the best binding site for the metal ions in the HA structure. In the low spin state, the ΔE of the HA-Fe(III), HA-Cu(II), and HA-Al(III) was 806.90, 566.92, 670.60 KJ/mol, respectively. HA-Cu(II) exhibiting the lowest ΔE demonstrated the weakest binding ability of Cu(II) to the HA, which was consistent with the results found with the stopped-flow spectrophotometer.

In summary, after metal incorporation, the role of HA as electron shuttles can be modified, enhanced or weakened, which is in agreement with changes in the quantity of electrons that can be reversibly transferred. Based on the results of microbial Fe(III) reduction, biocurrent generation and microbial azoreduction experiments, the magnitude of the accelerating effects or shuttling activities were in the order HA-Fe > HA-Cu > HA-Al. Because HA-M complexes commonly exist in the environment and because the effect of HA-M complexes on EET has rarely been studied, these findings have important implications for biogeochemical redox processes, given the ubiquity of both HA and various metals in the environment. In addition, because Fe-HA can promote extracellular electron transfer, this study provides a possible engineering application of HA-M complexes for bioremediation in soil.

## Methods

### Preparation of HA-M complexes

HA was purchased from Sigma-Aldrich (St. Louis, MO, USA).The Aldrich HA was purified using the method described by Monteil-Rivera *et al.*^32^. First, the HA was dissolved in 0.1 M NaOH, stirred for 6 h, and then centrifuged at 28000 × g to remove the insoluble humin. The supernatant was acidified to pH = 2 with 1 M HCl to precipitate the HA and separate it by centrifugation. The HA was washed several times with a mixture of 5 M HCl–5% HF to dissolve any remaining inorganic solids. The HA was then washed several times with 1.8 M HCl until the Fe content was minimized. The residue was thoroughly washed with deionized water to remove all chloride ions and gently dried at 40 °C. Metal salts, Fe(NO_3_)_3_·9H_2_O, CuCl_2_·2H_2_O and AlCl_3_·6H_2_O, were obtained from Shanghai Chemical Reagents (Shanghai, China) and were of analytical grade. HA-M complexes were prepared as described by Guardado *et al.*^33^. The procedure is described briefly as follows: Dissolve 10 g of HA in 50 ml of ultrapure water, stir the solution for 4 h, and use 1 M NaOH to maintain the pH at 10.5. Then, centrifuge the solution at 8000 rpm for 15 min. Dissolve 0.81 g of Fe(NO_3_)_3_·9 H_2_O (0.34 g of CuCl_2_·2H_2_O or 0.48 g of AlCl_3_·6H_2_O) in 2 ml of ultrapure water. Slowly add the solution dropwise into the supernatant containing HA, and adjust the pH to 8–9 with 1 M NaOH. Dilute the solution to 100 ml, stir for 14 h, and centrifuge at 8000 rpm for 15 min.

### Chemical analysis of HA-M complexes

Elemental analyses were performed on HA and freeze-dried HA-M complexes using an elemental analyzer (Elementar Analysensysteme GmbH Vario EL, HAMAU, Germany). Fe, Cu and Al concentrations in the HA and HA-M complex solutions were determined with inductively coupled plasma atomic emission spectrometry (ICP-AES, Perkin Elmer Optima 3000). The total organic C (TOC) concentration in the solutions was measured with a TOC digital reactor block (DRB200, HACH, USA) equipped with a spectrophotometer (DR2700, HACH, USA). Stock solutions of HA-M complexes were diluted to the desired concentrations before use. Freeze-dried samples were mixed with KBr at a ratio of 1:180 and compacted to form pellets. FTIR spectra were recorded between 4000 cm^−1^ and 400 cm^−1^ using a Hitachi EPI-G2 infrared spectrophotometer in transmission mode. XPS studies on freeze-dried samples were performed using an ESCALAB 250 (Thermo Fisher Scientific Inc., Waltham, MA, USA) with a monochromatic Al K*α* X-ray source (1486.6 eV) operated at 150 W. The base pressure in the system was 2 × 10^−9^ mbar. High-resolution spectra were collected using a pass energy of 20 eV^34^. The line shapes used for curve fitting were pure Gaussian. HA-M complexation kinetics were investigated with a stopped-flow spectrophotometer (Model SX20, Applied Photophysics Ltd., UK) equipped with a fluorescence detector at 25 °C in 0.01 M borate buffer (pH 6.5). Changes in the fluorescence emission resulting from complexation between HA (50 mg/L) and metal (1 × 10^−4^ M) were detected through a 395-nm cutoff filter (excitation at 350 nm).

### Electrochemical measurements of HA-M complexes

Cyclic voltammetry measurements were performed using an electrochemical workstation (CHI660D, Chenhua Co. Ltd, Shanghai, China)with a conventional three-electrode cell at ambient temperature with a potential range from 0.6 (vs. Ag/AgCl) to −0.8 V (vs. Ag/AgCl) as suggested by Yuan *et al.*^35^. The electron accepting capacity (EAC, measured at −0.6 V vs. Ag/AgCl) of the HA and HA-complexes was determined using a graphite plate (projected surface area of 17.5 cm^2^) working electrode, a Pt grid counter electrode, and a Ag/AgCl reference electrode, with 0.1 mol/l KCl as the supporting electrolyte. The determination of electron transfer reversibility was performed as described by Yuan *et al.*^36^ Briefly, the HA and HA-M complexes were first reduced at a potential of −0.6 V (vs. Ag/AgCl) and subsequently reoxidized at a potential of +0.5 V (vs. Ag/AgCl) with a total of 4 cycles.

### Microbial Fe(III) reduction mediated by HA-M complexes

A dissimilatory Fe(III) reduction experiment was conducted using serum bottles. Basal medium containing (per liter of deionized water) 0.68 g NaH_2_PO_4_·2H_2_O, 0.25 g NH_4_Cl, 0.10 g KCl, 10.0 ml vitamin stock solution, and 10.0 ml trace mineral solution^37^ was dispensed into the bottles. Lactate (final concentration of 5 mmol/l) and goethite (*α*-FeOOH, final concentration of 25 mmol/l) were added as electron donor and electron acceptor, respectively. The bottles except those for abiotic control were inoculated with *Shewanella oneidensis* MR-1. HA or HA-M complex (final concentration of 20 mg C/l) was added to the bottles, except those for biotic control. Standard anaerobic techniques were applied, and the utilized solutions and tools were strictly sterilized throughout the experiment. The bottles were incubated in the dark at 30 °C for 30 days. Every 5 days, three bottles were sacrificed for Fe(II) concentration determination. The total concentration of Fe(II), including dissolved and sorbed Fe(II), was determined by extracting Fe(II) from the samples using 0.5 mol/l HCl for 1.5 h^38^ and assaying the extract using the 1,10-phenanthroline colorimetric method^39^.

### Biocurrent generation mediated by HA-M complexes

Biocurrent was measured using a bioelectrochemical system as previously reported^40^. The measurements were performed under potentiostatic control (CHI660D, China) utilizing a three-electrode arrangement with a carbon cloth working electrode (7 cm^2^), a Ag/AgCl reference electrode and a Pt net counter electrode^41^. The bioelectrochemical reactor was filled with 20 ml of sterile minimal salt medium containing 10 mmol/l lactate and inoculated with *Shewanella oneidensis* MR-1 (1 × 10^7^ cfu/mL). HA or HA-M complex was added to the reactor to a final TOC of 20 mg C/l. Oxygen dissolved in the medium was bubbled out with ultrapure N_2_ prior to the addition.

### Microbial azoreduction mediated by HA-M complexes

20.0 ml basal medium and *Shewanella oneidensis* MR-1 at 1.0 × 10^7^ CFU/ml (OD_600_ = 1.0–1.2) were added to the serum bottles. Glucose at 5 mmol/l and azo dye Orange I at 0.5 mmol/l were added as an electron donor and electron acceptor, respectively. HA or HA-M complex was added to the bottles to a final TOC of 20 mg C/l, and for the blank, nothing was added. After purging with 100% N_2_ for 30 min, the samples were immediately sealed with butyl rubber bungs, crimped with aluminum caps and incubated in the dark at 30 °C. Samples were taken from the bottles at intervals and filtered through a 0.22-μm syringe filter (PVDF, Millipore Inc.). The absorbance at 515 nm, where Orange I displayed the maximum absorbance, was immediately determined with a spectrophotometer (TU1800-PC, Beijing). All treatments were conducted in triplicate. Strict anaerobic techniques were used throughout the experiment.

### Reaction rate constant calculations

This parameter was calculated as follows:


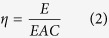


Here *EAC* (mol/g C) is the quantity of electrons accepting by the HA-Me. *E* (mol/g C) is calculated by different methods in different experiments. For the microbial Fe (III) reduction experiment, *E* is the difference value between the final and initial concentration of Fe(II). For the biocurrent generation experiment, *E* is the coulombs which calculated by integral area under the biocurrent curve. For the microbial azoreduction experiment, *E* is 4 e^−^ per mol difference between the initial and final concentration of the azo dye Orange I.

## Additional Information

**How to cite this article**: Zhou, S. *et al.* Influence of Humic Acid Complexation with Metal Ions on Extracellular Electron Transfer Activity. *Sci. Rep.* **5**, 17067; doi: 10.1038/srep17067 (2015).

## Supplementary Material

Supplementary Information
