# Supplementary material for: Influence of Humic Acid Complexation with Metal Ions on Extracellular Electron Transfer Activity
Source: Sci Rep. 2015 Nov 23;5:17067. doi: 10.1038/srep17067 (PMC4655413; doi:10.1038/srep17067)
Supplement: Supplementary Information [file srep17067-s1.doc]

**SUPPLEMENTARY DATA**

**Influence of Humic Acid Complexation with Metal Ions on Extracellular Electron Transfer Activity**

Shungui Zhou^1，*^,Shanshan Chen^1^,Yong Yuan^2^, Qin Lu^2^

^1^College of Resources and Environment, Fujian Agriculture and Forestry University, Fuzhou 350002, China

^2^Guangdong Institute of Eco-environmental and Soil Sciences, Guangzhou 510650, China

*Corresponding authors. E-mail: [sgzhou@soil.gd.cn](mailto:sgzhou@soil.gd.cn)；Tel: 86-591-86398906

Number of Pages (including this cover sheet): 9

Number of Figure: 5

Number of Table: 4

Number of scheme: 1

**Table S1 Elemental contents and Fe, Cu, and Al concentrations in HA-M** complexes

|  | **C (%)** | **O (%)** | **N (%)** | **S (%)** | **H (%)** | **Fe (mg/kg HA-Fe)** | **Cu (mg/kg HA-Cu)** | **Al (mg/kg HA-Al)** |
| --- | --- | --- | --- | --- | --- | --- | --- | --- |
| **HA** | 64.7 | 34.3 | 0.9 | 1.3 | 4.9 | \ | \ | \ |
| **HA-Fe** | 58.4 | 32.8 | 0.7 | 1.1 | 5.2 | 263.2 | \ | \ |
| **HA-Cu** | 59.5 | 33.2 | 0.8 | 1.2 | 5.1 | \ | 188.5 | \ |
| **HA-Al** | 59.1 | 33.6 | 0.8 | 1.3 | 5.2 | \ | \ | 239.4 |

**Table S2 FTIR peak intensity ratios of HA and HA-metal complexes**

|  | **1385 cm^-1^/3444 cm^-1^** | **1385 cm^-1^/1635cm^-1^** | **1385 cm^-1^/1141 cm^-1^** |
| --- | --- | --- | --- |
| **HA** | 2.742 | 1.404 | 1.170 |
| **HA-Fe** | 2.330 | 1.037 | 0.762 |
| **HA-Cu** | 2.194 | 1.292 | 1.305 |
| **HA-Al** | 2.109 | 1.286 | 1.169 |

**Table S3 Calculated energy of HA and various metal ions at low and high spin states**

| **Species** | **Spin state** | **Energy (a.u.)** |
| --- | --- | --- |
| **HA** | 1 | -2098.485087 |
| **Al^3+^** | 1 | -240.387380 |
| **Al^2+^** | 2 | -241.451775 |
| **Cu^2+^** | 2 | -195.829202 |
| **Cu^+^** | 1 | -195.829202 |
| **Fe^2+^** | 1 | -122.340138 |
|  | 5 | -122.480219 |
| **Fe^3+^** | 2 | -121.164076 |
|  | 6 | -121.297597 |

**Table S4 Bonding energy(KJ/mol) of the HA-M formed at various sites and different spin state**

| **Species** | **Site** | **Spin** | **E_HA-M_** | **ΔE** | **Spices** | **Spin** | **Ecomplexs** | **ΔE** |
| --- | --- | --- | --- | --- | --- | --- | --- | --- |
| Al^3+^ | a | 1 | -2339.912925 | 652.89 | Al^2+^ | 2 | -2340.392637 | 286.00 |
|  | b |  | -2339.907490 | 649.48 |  |  | -2340.374069 | 274.35 |
|  | c |  | -2339.884262 | 634.90 |  |  | -2340.359777 | 265.38 |
|  | d |  | -2339.941156 | **670.60** |  |  | -2340.398057 | **289.40** |
|  | e |  | -2339.886917 | 636.57 |  |  | -2340.349440 | 258.89 |
|  | f |  | -2339.249436 | 236.55 |  |  | -2340.325638 | 243.96 |
|  | g |  | -2339.360000 | 305.93 |  |  | -2340.312432 | 235.67 |
|  | h |  | -2339.290000 | 262.00 |  |  | -2340.328947 | 246.03 |
| Cu^2+^ | a | 2 | -2294.107888 | 350.11 | Cu^+^ | 1 | -2294.418634 | 65.48 |
|  | b |  | -2294.087126 | 337.08 |  |  | -2294.197214 | 73.46 |
|  | c |  | -2294.095515 | 342.35 |  |  | -2294.455530 | 88.63 |
|  | d |  | -2294.453405 | **566.92** |  |  | -2294.453405 | **87.30** |
|  | e |  | -2294.095534 | 342.36 |  |  | -2294.455496 | 88.61 |
|  | f |  | -2294.123710 | 360.04 |  |  | -2294.434653 | 75.53 |
|  | g |  | -2294.421379 | 546.83 |  |  | -2294.420379 | 66.57 |
|  | h |  | -2294.102755 | 346.89 |  |  | -2294.454770 | 88.15 |
| Fe^3+^ | a | 2 | -2220.896332 | 782.60 | Fe^2+^ | 1 | -2221.264046 | 275.36 |
|  | b |  | -2220.908276 | 790.09 |  |  | -2221.289678 | 291.44 |
|  | c |  | -2220.909351 | 790.77 |  |  | -2221.265023 | 275.97 |
|  | d |  | -2220.935060 | **806.90** |  |  | -2221.277612 | **283.87** |
|  | e |  | -2220.878036 | 771.12 |  |  | -2221.274161 | 281.71 |
|  | f |  | -2220.899016 | 784.28 |  |  | -2221.274161 | 281.71 |
|  | g |  | -2220.907025 | 789.31 |  |  | -2221.274161 | 281.71 |
|  | h |  | -2220.901036 | 785.55 |  |  | -2221.274161 | 281.71 |
|  | a | 6 | -2220.939516 | 725.91 |  | 5 | -2221.374796 | 256.95 |
|  | b |  | -2220.935869 | 723.62 |  |  | -2221.369119 | 253.39 |
|  | c |  | -2220.907679 | 705.93 |  |  | -2221.346296 | 239.07 |
|  | d |  | -2220.954084 | **735.05** |  |  | -2221.376203 | **257.84** |
|  | e |  | -2220.944204 | 728.85 |  |  | -2221.374687 | 256.89 |
|  | f |  | -2220.573669 | 496.34 |  |  | -2221.027425 | 38.98 |
|  | g |  | -2220.583695 | 502.63 |  |  | -2221.408877 | 278.34 |
|  | h |  | -2220.531627 | 469.96 |  |  | -2220.989605 | 15.25 |

**Scheme S1**

**(M=Al…)**

**(M=Fe, Cu…)**

**e**

**Electron acceptors**

**Electron acceptors**

**Electron acceptors**

**e**

**Microbe**

**Microbe**

**Microbe**

**HA**

**HA**

**HA**

**e**

**e**

**e**

**e**

**e**

**e**

**Scheme S1 Illustration of extracellular electron transfer mediated by HA and HA-M complex. Red arrows indicate electon flow direction and black arrows indicate redox process of the active sites. Crosses indicate that redox processes are inhibited.**

**Figure S1**

**Figure S1 FTIR spectra of the HA and HA-metal complexes.**

**Figure S2**

**Figure S2 High-resolution C 1s XPS spectra of the HA and HA-M complexes.**

**Figure S3**


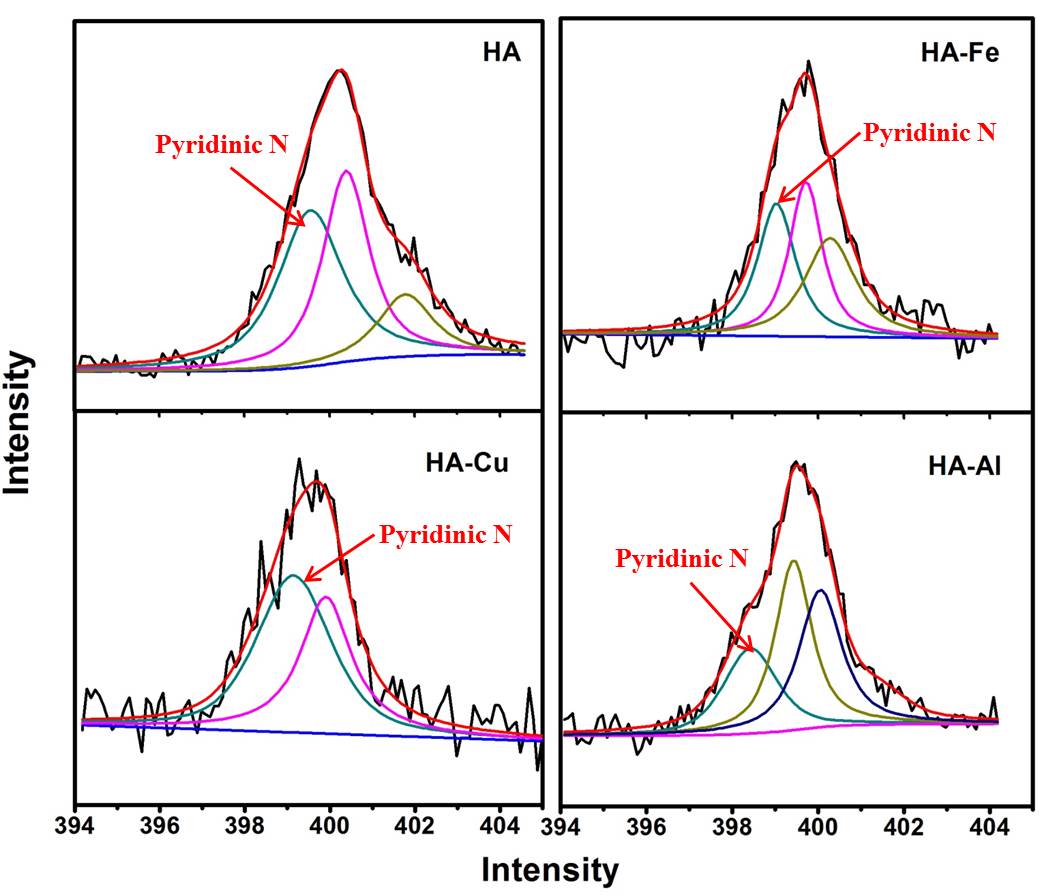


**Figure S3 High-resolution N 1s XPS spectra of the HA and HA-metal complexes.**

**Figure S4**

**Figure S4 High-resolution S 2p XPS spectra of the HA and HA-metal complexes.**

**Figure S5**

**
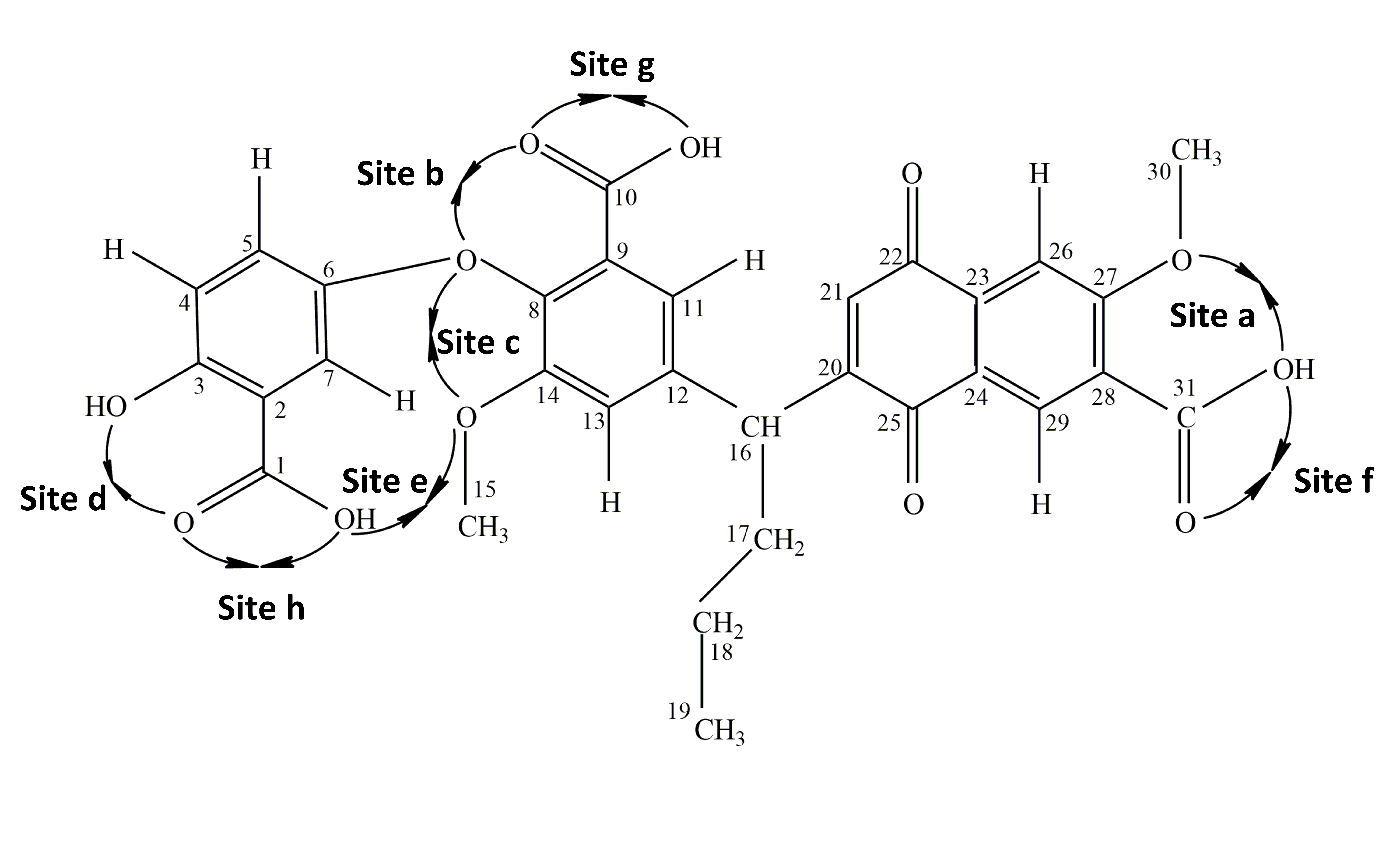
**

**Figure S5 The HA monomer including 8 possible active metal-binding sites (a-h).**
